# CYLD suppression enhances the pro-inflammatory effects and hyperproliferation of rheumatoid arthritis fibroblast-like synoviocytes by enhancing NF-κB activation

**DOI:** 10.1186/s13075-018-1722-9

**Published:** 2018-10-03

**Authors:** Le-meng Zhang, Jing-Jing Zhou, Chun-lei Luo

**Affiliations:** 1 Thoracic Medicine Department, Hunan Cancer Hospital, Changsha, 410013 People’s Republic of China; 2 Department of Rheumatology, Navy General Hospital, Beijing, 100048 People’s Republic of China; 3 Department of Nephrology, Ningbo First Hospital, Ningbo, 3150102 People’s Republic of China

**Keywords:** Rheumatoid arthritis (RA), Cylindromatosis (CYLD), Fibroblast-like synoviocyte (FLS), Nuclear factor κ-light-chain-enhancer of activated B cells (NF-κB), Pro-inflammatory cytokines, Hyperproliferation

## Abstract

**Background:**

Rheumatoid arthritis fibroblast-like synoviocytes (RA-FLSs) actively drive joint inflammation and degradation by producing inflammatory cytokines and matrix-degrading molecules, making them key factors in the pathogenesis of RA. Cylindromatosis (CYLD) is a tumor suppressor that downregulates nuclear factor kappa-light-chain-enhancer of activated B cells (NF-κB) activation by deubiquitinating NF-κB essential modulator and tumor necrosis factor receptor-associated factors 2 and 6. In this study, we aimed to determine CYLD expression in the synovium of patients with RA, analyze its correlation with NF-κB activation and clinical disease activity, further investigate CYLD expression in RA-FLSs, and explore CYLD’s roles and mechanisms in the pro-inflammatory effects, proliferation, apoptosis, and cell cycles of RA-FLSs.

**Methods:**

We obtained synovia from 50 patients with active RA and 20 with osteoarthritis (OA) and then cultured FLSs from the samples. We determined CYLD expression in the synovia of RA patients and in FLSs via reverse transcription polymerase chain reaction (RT-PCR). CYLD was depleted by lentiviral CYLD short hairpin ribonucleic acid. We used RT-PCR and enzyme-linked immunosorbent assay to analyze the expression of pro-inflammatory cytokines, matrix metalloproteinases (MMPs), and receptor activator of nuclear factor kappa-B ligand (RANKL). We detected cell proliferation using Cell Counting Kit-8 and examined cell apoptosis and cell cycle using flow cytometry.

**Results:**

We obtained the following results:

1. In synovia from patients with RA, CYLD expression was significantly downregulated while NF-κB expression was distinctly upregulated, compared with synovia from patients with OA. Thus, there is a significant inverse correlation between CYLD and NF-κB in synovia affected by RA.
2. CYLD expression significantly decreased in RA-FLSs compared with OA-FLSs.
3. CYLD suppression enhanced the production of pro-inflammatory cytokines, MMPs, and RANKL by activating NF-κB in RA-FLSs.
4. CYLD suppression enhanced proliferation, reduced apoptosis, and increased cell division of RA-FLSs and aggravated the activity of NF-κB in RA-FLSs.

**Conclusions:**

Via its regulation of NF-κB activation, CYLD may be involved in the pathogenesis of synovial inflammation in RA as well as in the pro-inflammatory effects and hyperproliferation of RA-FLSs. CYLD may therefore provide a potential target for the treatment of RA.

## Background

Rheumatoid arthritis (RA) is a systemic and chronic inflammatory disease characterized by synovial hyperplasia formation, which mediates cartilage and bone destruction [1].The pathogenesis of RA is extraordinarily complicated and involves various kinds of cells, such as fibroblast-like synoviocytes (FLSs), T cells, B cells, monocytes/macrophages, and osteoclasts [2]. It has been indicated that each cell type plays distinct, complex, and interrelated roles in the development of RA [3]. In RA, the synovial lining thickness at the border of the joint cavity increases to 10–15 cell layers, compared with 1–3 cell layers in normal individuals [4, 5]. Prior studies have demonstrated that FLSs are the predominant cell type in the terminal layer of the hyperplastic synovium and at the sites of invasion in adjacent cartilage and bone joints, and that they vigorously contribute to the initiation and early perpetuation of RA [4, 6].

FLSs in RA, also known as synovial fibroblasts or type B synoviocytes, occur early after onset with visible erosion at cartilage–bone junctions [7]. FLS activation involves multiple factors, including Toll-like receptor (TLR) activation, inflammatory factors, matrix degradation products, and epigenetic modifications [8]. Activated RA-FLSs are highly able to recruit, retain, and activate both cells of the immune system and resident joint cells through hyperproduction of a broad array of pro-inflammatory cytokines, matrix metalloproteinases (MMPs), and chemokines, leading to the initiation and maintenance of chronic inflammation and progressive joint destruction [9]. The pro-inflammatory cytokine tumor necrosis factor alpha (TNF-α) is regarded as one of the most central cytokines that significantly triggers inflammation and joint destruction, which is confirmed by the clinical efficacy of TNF-α-blocking agents [10]. TNF-α, interleukin-1β (IL-1β), interleukin-6 (IL-6), and interleukin-8 (IL-8), the most important cytokines, can obviously aggravate inflammatory reactions and increase the influx of additional pro-inflammatory cells into the synovium to sustain regulatory feedback loops and induce production of MMPs, cathepsins, and aggrecanases [11, 12]. The synovium is the main source of MMPs and cathepsins in RA, and in situ hybridization studies have localized collagenase messenger ribonucleic acid (mRNA) nearly exclusively to RA-FLSs [13]. Activated MMPs can significantly drive the degradation of the extracellular matrix (ECM) and facilitate cartilage and bone erosion, while inhibition of MMPs can greatly alleviate the invasiveness of RA-FLSs into cartilage [14–16]. RA-FLSs also influence bone destruction via modulation of osteoclastogenesis by secreting receptor activator of nuclear factor kappa-light-chain-enhancer of activated B cells (NF-κB) ligand (RANKL) [17]. Denosumab, a human monoclonal antibody that specifically binds RANKL, has been effective for the treatment of RA-associated bone loss [18, 19]. In addition, synovial hyperplasia in RA appears to be caused at least in part by the impairment of apoptosis in RA-FLSs and synovial macrophages, while deficient apoptosis has been shown to prolong RA-FLS survival by increasing the production of anti-apoptotic molecules like B-cell lymphoma 2 (Bcl-2), sentrin-1 (SUMO-1), and Fas-associated death domain-like interleukin-1- converting enzyme inhibitory protein (FLIP) [19, 20].

Cylindromatosis (CYLD) was initially identified as a tumor suppressor that is mutated in patients with familial cylindromatosis, a genetic condition that predisposes patients for the development of skin appendage tumors (cylindroma) [21]. CYLD is a key adaptor that regulates various signaling pathways to modulate diverse physiological processes, ranging from immune response and inflammation to cell cycle progression, spermatogenesis, and osteoclastogenesis [22]. Emerging evidence suggests that CYLD primarily downregulates NF-κB signaling by deubiquitinating NF-κB essential modulator (NEMO) and several upstream regulators like TNF receptor-associated factors 2 and 6 (TRAF2 and TRAF6) and TGF-β-activated kinase 1 (Tak1) [23–25]. The family of NF-κB transcription factors plays a large role in mediating the expression of large numbers of genes during the inflammatory response. Many previous in vitro and in vivo studies on the contribution of components of NF-κB signaling pathways to the pathogenesis of RA have shown that NF-κB plays prominent roles in inflammation, cartilage degradation, cell proliferation, angiogenesis, and pannus formation [26]. Until now, however, the role and the underlying mechanism of CYLD in local inflammation and joint destruction in RA have been poorly elucidated. Therefore, in this study, we determined CYLD expression in synovia from patients with RA and analyzed its correlation with NF-κB activation or clinical disease activity. Then we investigated CYLD expression in RA-FLSs and explored its roles and mechanisms in pro-inflammatory effects, proliferation, apoptosis, and cell cycles of RA-FLSs.

## Methods

### Patients

We recruited 50 Chinese patients with RA who fulfilled the 1987 revised criteria of the American College of Rheumatology (ACR) for RA [27] or the 2010 ACR/European League Against Rheumatism (EULAR) classification criteria for RA [28] from the Department of Rheumatology and Orthopedics. All RA patients had a Disease Activity Score 28-joint assessment (DAS28) ≥ 3.0 (active disease). We obtained the names of 20 patients with osteoarthritis (OA) who fulfilled established clinical criteria as “less-inflamed” disease controls from the Department of Rheumatology and Orthopedics [29, 30]. This study was conducted in compliance with the Helsinki Declaration, and the protocol was approved by the Ethics Committee of Navy General Hospital, Beijing, China. All patients gave written informed consent.

### Disease assessments

Clinical data of all patients with RA were collected at baseline, including tender joint count of 28 joints (28TJC), swollen joint count of 28 joints (28SJC), patient and provider global assessment of disease activity (PtGA and PrGA, respectively), pain visual analog scale (pain VAS), Chinese-language version of Stanford Health Assessment Questionnaire (HAQ) [31], erythrocyte sedimentation rate (ESR), C-reactive protein (CRP), rheumatoid factor (RF), and anti-cyclic citrullinated peptide antibody (ACPA). We assessed disease activity with DAS28 using 4 variables, including CRP (DAS28 [4]-CRP) [32].

### Synovial tissue collection

We collected the synovium from patients with RA via biopsy with a closed Parker Pearson needle [33]. We obtained at least six pieces of synovial tissue per patient to minimize sampling error [34]. The synovia were obtained from the knees of OA patients by closed-needle biopsy, arthroplasty, or arthroscopy. We fixed all samples in 10% neutral formalin and embedded them in paraffin. Sections (5 μm) were cut serially and mounted on adhesive glass slides. The sealed slides were stored at − 20 °C until staining.

### Immunohistochemistry (IHC)

We stained serial sections of synovial tissues with hematoxylin and eosin (H&E) and a three-step immunoperoxidase method for IHC. Sections were incubated with CYLD (Abcam, Cambridge, MA, USA) at 1/100 dilution overnight at 4 °C after deparaffinization and retrieval, then with EnVision mouse or rabbit conjugate (Dako/Agilent, Santa Clara, CA, USA) for 30 min at 37 °C. We used 3,3′-diaminobenzidine (DAB)-positive substrate for the color reaction, and we counterstained sections with hematoxylin. We used nonspecific isotype immunoglobulin G (IgG) as a negative control. We determined the percentage of CYLD-positive staining cells in the lining layers and sublining area by manually observing five different fields at × 400 magnification.

### Fibroblast-like synoviocyte (FLS) culture

We isolated FLSs from the synovial tissues using the modified tissue culture method [35], shredded fresh synovial tissues into small pieces, and digested them in type I collagenase (Sigma-Aldrich, St. Louis, MO, USA) for 2 h at 37 °C. The cells were cultured with complete Dulbecco’s modified Eagle’s medium–Ham’s F-12 (DMEM/F12; Gibco Life Technologies, Shanghai, China) containing 100 g/ml streptomycin, 100 units/ml penicillin, and 20% fetal bovine serum (FBS; Gibco Life Technologies, Australia) in a humidified 5% CO_2_ incubator. Our in vitro study used FLSs from passages 3–5.

### Immunofluorescence (IF) staining of FLSs

After fixation and permeabilization, we blocked the FLSs with 5% bovine serum albumin (BSA), then incubated the cells in phosphate-buffered saline (PBS) containing rabbit anti-human polyclonal antibody to CYLD (Abcam, USA) or normal rabbit IgG (control) overnight at 4 °C. The secondary antibody (red) was Alexa Fluor 633 conjugated goat anti-rabbit IgG (Invitrogen, Carlsbad, CA, USA), used at a 1:1000 dilution for 1 h at 37 °C. We used 4,′6-diamindino-2-phenylindole (DAPI; Sigma-Aldrich) to stain the cell nuclei (blue) at a concentration of 1.43 μM for 3 min and mounted ProLong Gold Antifade Reagent (P36934; Invitrogen) to the coverslips. Images were examined and analyzed with a 160 Zeiss LSM 510 confocal microscope (Carl Zeiss AG, Jena, Germany).

### Lentivirus infection in RA-FLSs

We performed gene silencing with lentiviral short hairpin RNA (shRNA). The sh-CYLD targeting sequence was GCCCAATACCAATGGAAGTAT. We cloned shRNA into pLKO.1 (gv248) lentiviral vectors, added culture supernatants containing shRNA to RA-FLSs in the presence of polybrene, and selected the cells using 1 μg/ml puromycin after 24 h. Stable cell lines were verified by reverse transcription polymerase chain reaction (RT-PCR) and Western blot.

### Reverse transcription polymerase chain reaction (RT-PCR)

We isolated total RNA from the synovium or cells using RNAiso Plus (Takara Bio, Inc., Kusatsu, Japan) per the manufacturer’s instructions and synthesized complementary DNA (cDNA) samples using a reverse transcription kit (PrimeScript RT Master Mix; Takara Bio). We amplified the cDNA using specific oligonucleotide primers (Table 1). We performed quantitative real-time PCR (RT-qPCR) using SYBR Premix Ex Taq (Tli RNase H Plus; Takara Bio). The reactions were initiated with denaturation of cDNA templates at 95 °C for 30 s, 95 °C for 5 s, and 60 °C for 30 s, then amplification for 40–50 cycles. We ran samples in triplicate in a Roche LightCycler 480 sequence detection system (Roche, Basel, Switzerland) per the manufacturer’s instructions. All primers used in this study were synthesized by Invitrogen Genetech Co. Ltd. (Shanghai, China). As a control for sample loading, we performed PCR amplification of the housekeeping gene *β-actin* for each sample. We normalized and quantified PCR signals by comparing the cycle threshold value of the gene in question, in triplicate, with *β-actin*. Each bar represents mean ± standard deviation (SD) of three independent experiments was showed in Figs. 3 and 4.

**Table 1:** Primers for reverse transcription-polymerase chain reaction (RT-PCR)

β-actin	Sense: 5’-GGACTTCGA GCAAGAGATGG-3′
	Antisense: 5’-TGTGTTGGCGTACAGGTCTTTG-3’
NF-κB	Sense: 5’-ATGTGGAGATCATTGAGCAGC-3’
	Antisense: 5’-CCTGGTCCTGTGTAGCCATT-3’
CYLD	Sense: 5’-ACGCCACAATCTTCATCACACT-3’
	Antisense: 5’-AGGTCGTGGTCAAGGTTTCACT-3’
TNF-α	Sense: 5’-GCTAAGAGGGAGAGAAGCAACTACA-3’
	Antisense: 5’-GAAGAGGCTGAGGAACAAGCA-3’
IL-6	Sense: 5’-CTGCGCAGCTTTAAGGAGTTC-3’
	Antisense: 5’-CAATCTGAGGTGCCCATGCTA-3’
IL-1β	Sense: 5’-CCAGCTACGAATCTCCGACC-3’
	Antisense: 5’-CATGGCCACAACAACTGACG-3’
IL-8	Ssense: 5’-GTGCAGAGGGTTGTGGAGAAGTTT-3’
	Antisense: 5’-TCACTGGCATCTTCACTGATTCTTG-3’
MMP-1	Ssense: 5′- AAAATTACACGCCAGATTTGCC-3’
	Antisense: 5′- GGTGTGACATTACTCCAGAGTTG-3’
MMP-3	Sense: 5′- TTTCCAGGGATTGACTCAAAGA-3’
	Antisense: 5′- AAGTGCCCATATTGTGCCTTC-3’
RANKL	Sense: 5’-ACCAGCATCAAAATCCCAAG-3’
	Antisense: 5’-CCCCAAAGTATGTTGCATCC-3’

### Western blot analysis

After transfection, we separated cytoplasmic protein from nuclear protein using NE-PER nuclear and cytoplasmic extraction reagents (Thermo Scientific–Pierce, Rockford, IL, USA). We measured protein concentrations by bicinchoninic acid (BCA) protein assay (Pierce, Rockford, IL, USA). Protein (50 μg) was loaded onto 12% gradient sodium dodecyl sulfate- polyacrylamide gel electrophoresis (SDS-PAGE) gel under denaturing conditions and electrotransferred to nitrocellulose membranes. We blocked the blots with 5% milk in Tris-buffered saline-Tween (TBST) for 1 h at room temperature, then probed them with antibodies against CYLD (Abcam), p-nuclear factor of κ-light polypeptide gene enhancer in B-cells inhibitor-α (IκBα), T-IκBα, p-NF-κB p65 (Ser536), and NF-κB p65 (all CST (Cell Signaling Technology, Inc), USA; all 1:1000), glyceraldehyde 3-phosphate dehydrogenase (GAPDH; CST; 1:1000) and Histone H3 (CST; 1:2000) at 4 °C overnight. After washing them with TBST, we incubated the membranes with horseradish peroxidase (HRP)-conjugated secondary antibody (EarthOx Life Sciences, Millbrae, CA, USA) at 1:5000 dilution for 1 h at room temperature. We visualized immunoreactive bands by enhanced chemiluminescence (ECL; Amersham Pharmacia Biotech, Little Chalfont, UK) reaction. We conducted densitometric analyses on protein bands using a scanning imager, ImageQuant TL software (GE Healthcare Life Sciences) and the G:BOX Gel & Blot Imaging Series (Syngene, Cambridge, UK) and normalized the bands against the intensity of Histone H3 or GAPDH, expressing it as a fold change relative to untreated controls. Each blot is representative of at least three similar independent experiments.

### Enzyme-linked immunosorbent assay (ELISA)

We used ELISA (R&D Systems, Minneapolis, MN, USA) per the manufacturer’s instructions to detect the amounts of TNF-α, IL-1β, IL-6, MMP-1, MMP-3, and RANKL in the culture supernatants of the stably transduced RA-FLSs. Briefly, we added the culture supernatant mixed with assay buffer to wells coated with anti-TNF-α, IL-1β, IL-6, MMP-1, MMP-3, and RANKL antibody at 37 °C for 60 min. We then added HRP-conjugated anti-human TNF-α, IL-1β, IL-6, MMP-1, MMP-3, and RANKL monoclonal antibody and incubated at 37 °C for 2 h, followed by incubation with colorimetric (tetramethylbenzidine) solution for another 10 min. After that, we measured relative absorbance, and three independent experiments were performed for each condition.

### Cell proliferation assay

We seeded stably transduced RA-FLSs at a density of 5000 cells/well of each group in 96-well microtiter plates, then pre-incubated them in a humidified atmosphere with 5% CO_2_ at 37 °C. We added 10 μl of Cell Counting Kit-8 (CCK-8; Dojindo Molecular Technologies, Inc., Kyushu, Japan) solution to each well and incubated the RA-FLSs at 37 °C for 2 h at various time points (0, 24, 48, and 72 h after initial seeding). We used a microplate reader to detect absorbance at wavelength 450 nm (450 optical density [OD]). All experiments were conducted in triplicate.

### Apoptosis detection assay and cell cycle analysis by flow cytometry (FCM)

To study the effects of CYLD on RA-FLS apoptosis, we used sodium nitroprusside (SNP; Sigma-Aldrich) to induce apoptosis of stably transduced RA-FLSs. We measured apoptosis by detecting allophycocyanin (APC)-conjugated annexin-V and 7-aminoactinomycin D (7-AAD) using FCM (BD Biosciences Pharmingen, San Diego, CA, USA). For cell cycle analysis, we fixed and permeabilized the RA-FLSs with cold ethanol overnight, then treated them with propidium iodide (PI) and RNA enzyme (RNase) before subjecting them to fluorescence-activated cell sorting (FACS) analysis. We determined cell cycle distribution and quantification using FCM and CellQuest software (BD Biosciences, Mountain View, CA, USA). Each condition was repeated independently three times.

### Statistical analysis

We performed statistical analysis using SPSS for Windows statistical software version 13.0 (SPSS Inc., Chicago, IL, USA). Data are presented as mean ± standard deviation (SD) of three independent experiments. We used one-way analysis of variance (ANOVA) to compare data among groups and Bonferroni’s test for post hoc comparison. To assess the correlation between synovial expression of CYLD and NF-κB and that between CYLD expression in RA-FLSs and histological or clinical parameters, we used Spearman’s rank-order correlation. A *P* value < 0.05 was considered statistically significant.

## Results

### Characteristics of the study patients

Table 2 shows baseline demographic and clinical characteristics of all RA patients. Age and gender did not differ between patients with RA and those with OA. Thirty-eight of the RA patients were female and 12 were male. The mean age was 60 (range 49–64) years. The mean disease duration was 38 (range 14–110) months. Of the RA patients, 92% (46/50) had positive RF, while 82% (41/50) were positive for ACPA. All RA patients had DAS28 [4]-CRP values > 3.0. The mean DAS28 [4]-CRP score was 5.02 (range 3.0–6.8). Among the patients with RA, 54% (27/50) had never been treated with corticosteroids or disease-modifying anti-rheumatic drugs (DMARDs). Most of them had taken only Chinese herbals and/or painkillers to relieve arthralgia. A total of 16% (8/50) had taken corticosteroids alone before being referred to our hospital. A total of 30% (15/50) accepted one or more DMARDs, including methotrexate, leflunomide, sulfasalazine, hydroxychloroquine, or etanercept.

**Table 2:** Baseline demographic and clinical features of RA patients in the study

Characteristic	RA patients (*n* = 50)
Demographic	
Age, years, median (IQR)	60 (49~ 64)
Female, *n* (%)	38 (76)
Disease status	
Disease duration, months, median (IQR)	38 (14–110)
ESR (mm/h), median (IQR)	80 (54~ 105)
CRP (mg/dl), median (IQR)	4.37 (1.82~ 6.75)
Rheumatoid factor-positive, *n* (%)	46 (92)
ACPA-positive, *n* (%)	41 (82)
DAS28, median (IQR)	5.02 (3.0~ 6.8)
Previous medications, *n* (%)	
Corticosteroids	19 (38)
Methotrexate	20 (40)
Leflunomide	6 (12)
Sulfasalazine	2 (4)
Hydroxychloroquine	7 (14)
Etanercept	5 (10)

*RA* rheumatoid arthritis, *IQR* interquartile range, *ESR*, erythrocyte sedimentation rate, *CRP*, C-reactive protein, *ACPA*, anti-cyclic citrullinated peptide antibody, *DAS28*, Disease Activity Score 28-joint assessment, *n*, number of patients, *SD*, standard deviation

### Synovial CYLD and NF-κB expression in RA and OA

**Fig. 1:**
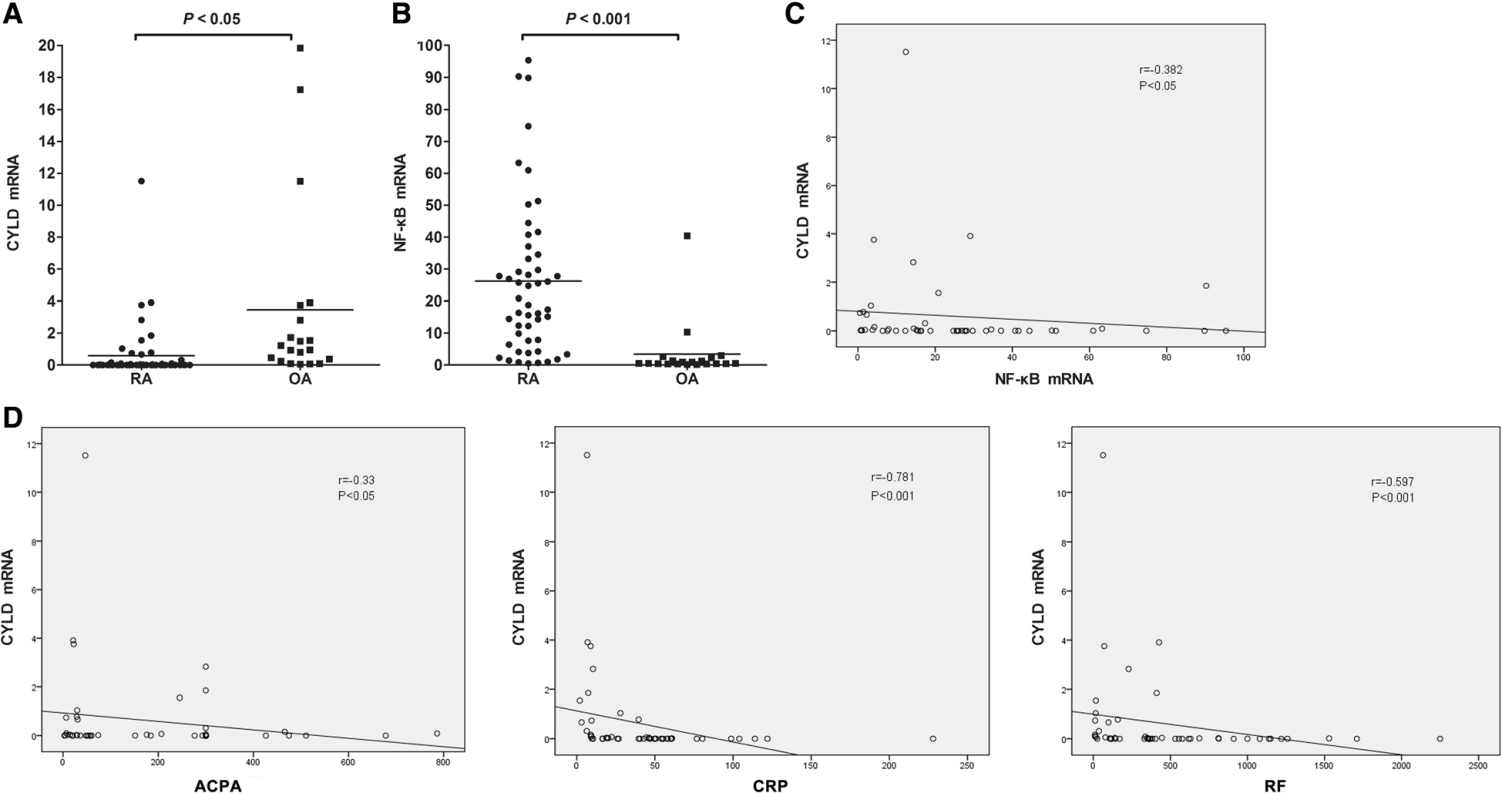
Synovial CYLD expression and its correlation with synovial NF-κB expression and clinical parameters. **a** Synovial CYLD expression in RA and OA. **b** Synovial NF-κB expression in RA and OA. **c** Correlation of synovial CYLD expression with NF-κB expression. **d** Correlation of synovial CYLD expression with clinical parameters. Data are represented as mean ± SD

The following results were obtained by RT-PCR analysis of synovia from 50 RA patients and 20 OA patients. Figure 1a shows that CYLD mRNA expression in synovia from RA patients was significantly decreased compared with that in synovia from OA controls (0.59 ± 1.81 vs. 3.46 ± 5.77; *P* < 0.05). Figure 1b indicates that NF-κB mRNA expression in synovia from RA patients was obviously increased compared with that in synovia from OA controls (26.17 ± 24.36 vs. 3.42 ± 9.00; *P* < 0.001).

### Correlation of synovial CYLD expression with NF-κB expression

To find the association between CYLD and NF-κB, we examined the correlation between CYLD and NF-κβ mRNA expression in synovia from RA patients. Using Spearman’s test, we found a significant inverse correlation between CYLD and NF-κβ mRNA expression in synovia from RA patients (Fig. 1c; *r* = − 0.382, *P* < 0.01).

### Correlation of synovial CYLD expression with clinical parameters

Spearman’s test showed conspicuous correlations between synovial CYLD mRNA expression and ACPA, CRP and RF (Fig. 1d; *r* = − 0.330, *P* < 0.05; *r* = − 0.781, *P* < 0.001; *r* = − 0.597, *P* < 0.001, respectively). There was no significant correlation between CYLD mRNA and 28TJC, 28SJC, PtGA, PrGA, HAQ, ESR, Simple or Clinical Disease Activity Index (SDAI and CDAI, respectively) or DAS28 (all *P* > 0.05).

### Expression of CYLD in RA-FLSs

**Fig. 2:**
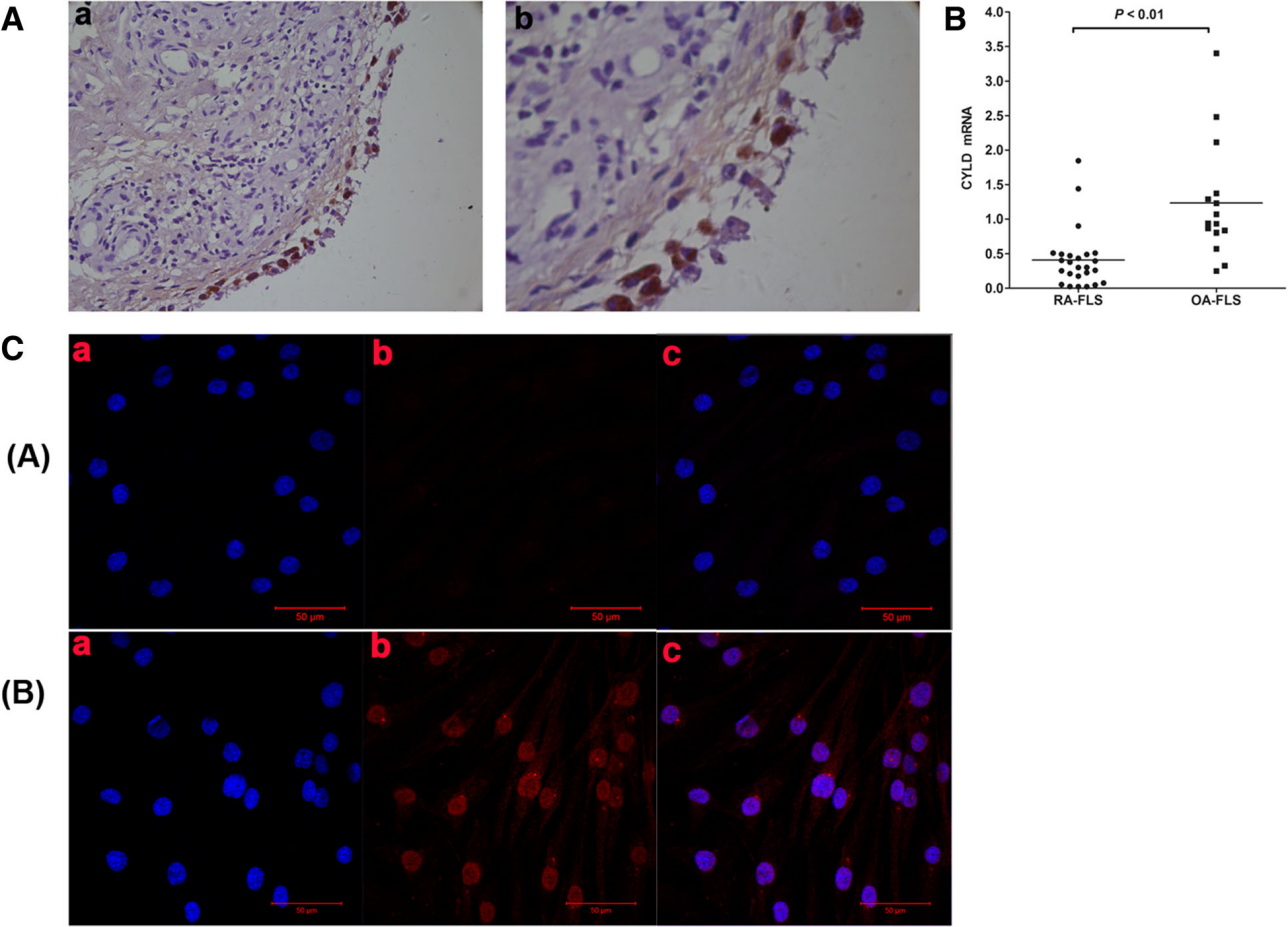
IHC and IF staining showing CYLD expression in primary cultures of FLSs from OA and RA patients. (**A**) Representative IHC findings of synovial CYLD expression. **a**, × 100; **b**, × 400. (**B**) CYLD mRNA expression in FLSs from RA patients (*n* = 25) compared with those from OA patients (*n* = 15), evaluated by RT-PCR. (**C**) IF staining of CYLD in primary cultures of FLSs from OA and RA patients. ([**A**] RA-FLSs, [**B**] OA-FLSs; **a**, DAPI [*blue*]; **b**, CYLD [*red*]; **c**, merged **a** and **b**. **a**, **b**: original magnification × 400.) Data are represented as mean ± SD

We observed CYLD expression in the lining and sublining layer of synovium from RA patients, finding intense staining in the endochylema as well as the nuclei of lining cells (both macrophage-like synoviocytes and FLSs) and sublining inflammatory cells (mostly in lymphocytes and plasma cells; Fig. 2A). To further investigate whether CYLD expression was aberrant in FLSs, we determined CYLD expression in 25 RA-FLSs and 15 OA-FLSs. Via RT-PCR we found that expression of CYLD mRNA in RA-FLSs was significantly lower than in OA-FLS controls (Fig. 2B; 0.41 ± 0.43 vs. 1.23 ± 0.84; *P* < 0.01), and via IF staining we also found that expression of CYLD in RA-FLSs was obviously lower than in OA-FLSs (Fig. 2C).

### shRNA transfection suppressed CYLD expression in RA-FLSs

**Fig. 3:**
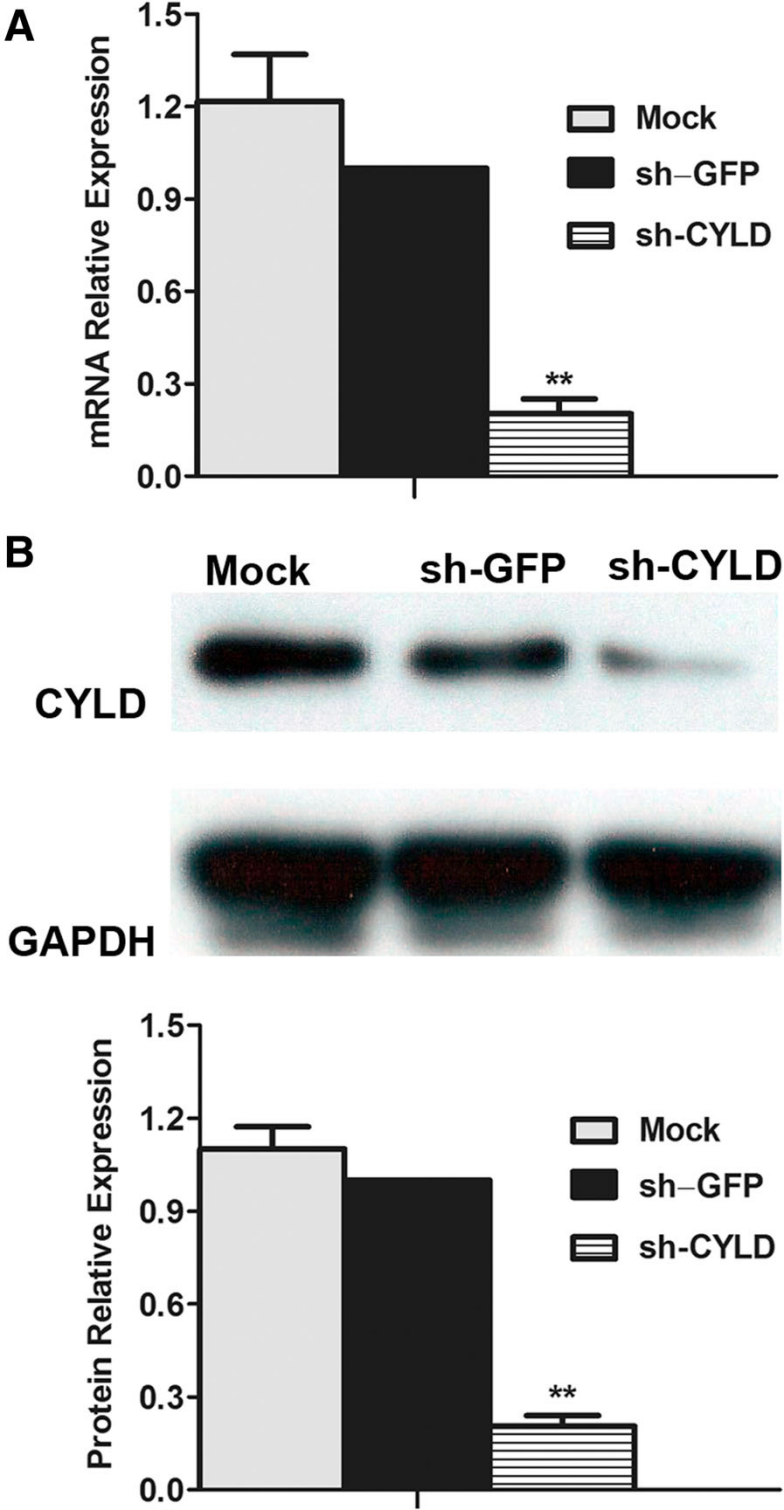
Effect of shRNA transfection on CYLD expression in RA-FLSs. We transfected RA-FLSs with sh-CYLD over 48 h. **a** We evaluated mRNA level of CYLD in FLSs by RT-PCR. **b** We detected the protein level of CYLD by Western blot. Data are represented as mean ± SD from 3 independent experiments. **P < 0.01

To investigate whether the constitutive expression of CYLD in RA-FLSs could be decreased by lentiviral CYLD shRNA (sh-CYLD), we transfected RA-FLSs with sh-CYLD or lentiviral vector (sh-GFP). RT-PCR showed that expression of CYLD in mRNA levels was remarkably downregulated in RA-FLSs transfected with sh-CYLD, compared with those transfected with sh-GFP (Fig. 3a; 0.20 ± 0.04 vs. 1.00 ± 0.00; *P* < 0.01). Western blot indicated similar results for protein levels (Fig. 3b; 0.21 ± 0.03 vs. 1.00 ± 0.00; *P* < 0.01).

### CYLD knockdown enhanced the pro-inflammatory effects of RA-FLSs

**Fig. 4:**
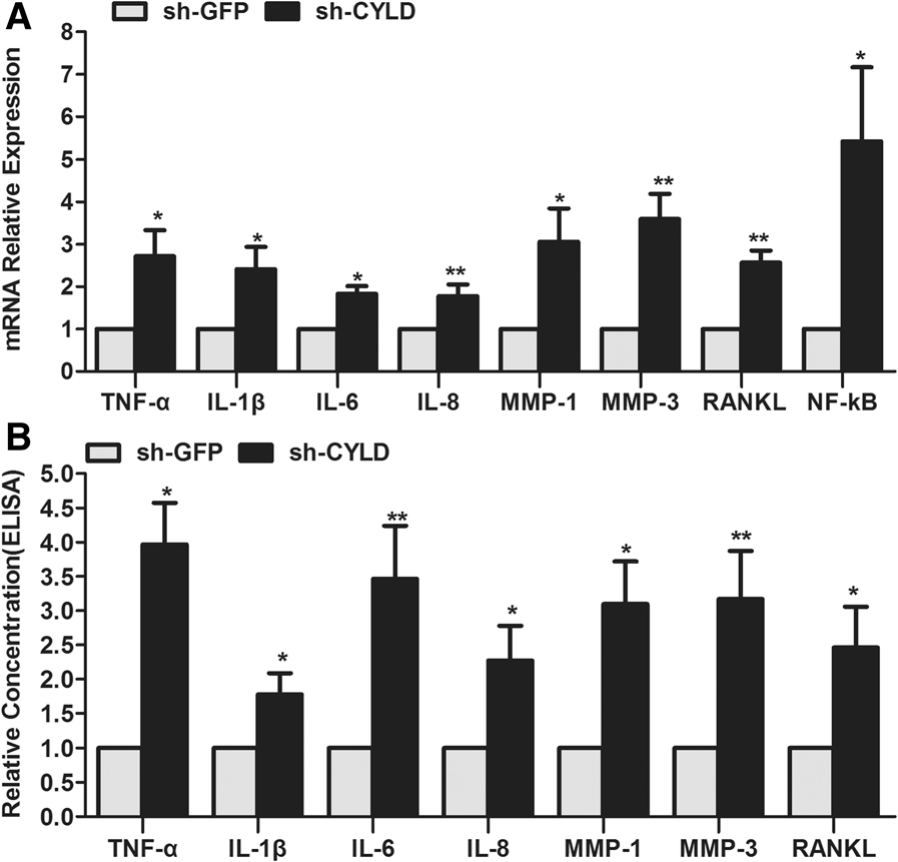
CYLD knockdown enhances the production of pro-inflammatory cytokines, MMPs, RANKL, and NF-κB in RA-FLSs. **a** After CYLD knockdown, we examined pro-inflammatory cytokines, MMPs, and RANKL mRNA expression in FLSs by RT-PCR. **b** After CYLD knockdown, we evaluated the protein level of pro-inflammatory cytokines, MMPs, and RANKL by ELISA. Data are represented as mean ± SD from 3 independent experiments. *P < 0.05, **P < 0.01

To further determine the role CYLD plays in the pro-inflammatory effects of RA-FLSs, we compared pro-inflammatory cytokine production in CYLD-knockdown RA-FLSs with that in RA-FLSs infected with sh-GFP. After sh-CYLD transfection, not only was mRNA expression of pro-inflammatory cytokines such as TNF-α, IL-1β, IL-6, and IL-8 strengthened, but that of MMP-1, MMP-3, RANKL, and NF-κB was also obviously enhanced compared with sh-GFP transfection (Fig. 4a). Similarly, protein levels of TNF-α, IL-1β, IL-6, IL-8, MMP-1, MMP-3, and RANKL in the cell culture supernatants of sh-CYLD-transfected RA-FLSs were markedly higher than those in supernatants of sh-GFP-transfected RA-FLSs (Fig. 4b).

### CYLD knockdown enhanced RA-FLS proliferation

**Fig. 5:**
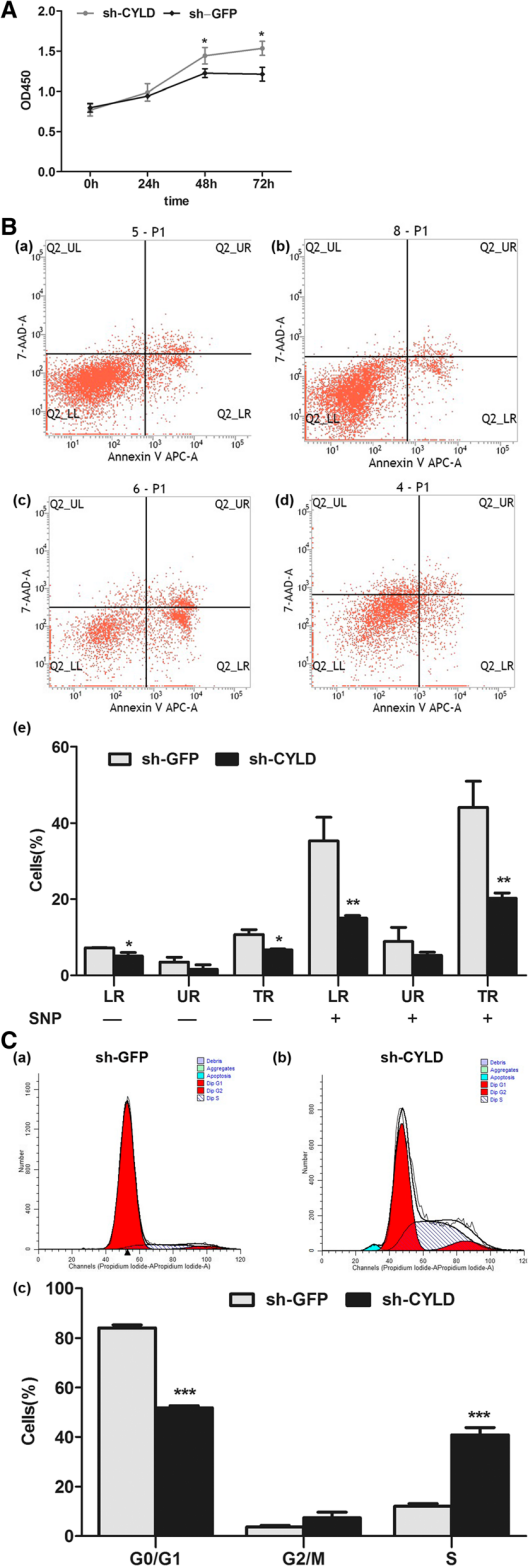
Effects of CYLD on cell proliferation, apoptosis, and cell cycle in RA-FLSs. We transfected RA-FLSs with sh-CYLD or sh-GFP. (**A**) CCK-8 test showed increased RA-FLSs proliferation in the sh-CYLD transfection group compared with the sh-GFP transfection group. (**B**) FCM analysis demonstrating the effect of CYLD suppression on cell apoptosis. (**C**) FCM analysis demonstrating the effect of CYLD suppression on cell cycle progression. Data are represented as mean ± SD from 3 independent experiments. *P < 0.05, **P < 0.01, ***P < 0.001

CCK-8 test results showed increased RA-FLS proliferation in sh-CYLD transfection groups compared with sh-GFP transfection groups (Fig. 5A). FCM analyses of annexin-V, APCA, and PI revealed that lentivirus-mediated inhibition of CYLD significantly prevented early and total apoptosis (LR and TR, respectively) but not late apoptosis (UR) when compared with the sh-GFP groups (Fig. 5B; LR 4.98 ± 0.94 vs. 7.15 ± 0.10, *P* = 0.016; UR 1.65 ± 1.14 vs. 3.51 ± 1.19, *P* = 0.122; TR 6.63 ± 0.21 vs. 10.67 ± 1.28, *P* = 0.029). The data also showed that CYLD knockdown markedly mitigated LR and TR but not UR in the presence of SNP, compared with the sh-GFP groups (Fig. 5B; LR 14.95 ± 0.71 vs. 35.30 ± 6.20, *P* = 0.005; UR 5.22 ± 0.78 vs. 8.80 ± 3.74, *P* = 0.236; TR 20.17 ± 1.41 vs. 44.10 ± 6.80, *P* = 0.004). The percentage of cells in G0/G1 phase was significantly decreased in sh-CYLD-transfected RA-FLSs compared with the sh-GFP groups (Fig. 5C; 51.80 ± 0.83 vs. 84.15 ± 1.18; *P* = 0.000). Conversely, the percentage of cells in S-phase was apparently enhanced in sh-CYLD-transfected RA-FLSs compared with the sh-GFP groups (Fig. 5C; 40.83 ± 2.92 vs. 12.10 ± 1.02; *P* = 0.000). Annexin-V assay results showed that the percentage of cells in G2/M phase was not significantly different between the sh-CYLD and the sh-GFP transfection groups (Fig. 5C; 7.38 ± 2.26 vs. 3.75 ± 0.51; *P* = 0.053).

### CYLD knockdown aggravated NF-κB and IκBα activation in RA-FLSs

**Fig. 6:**
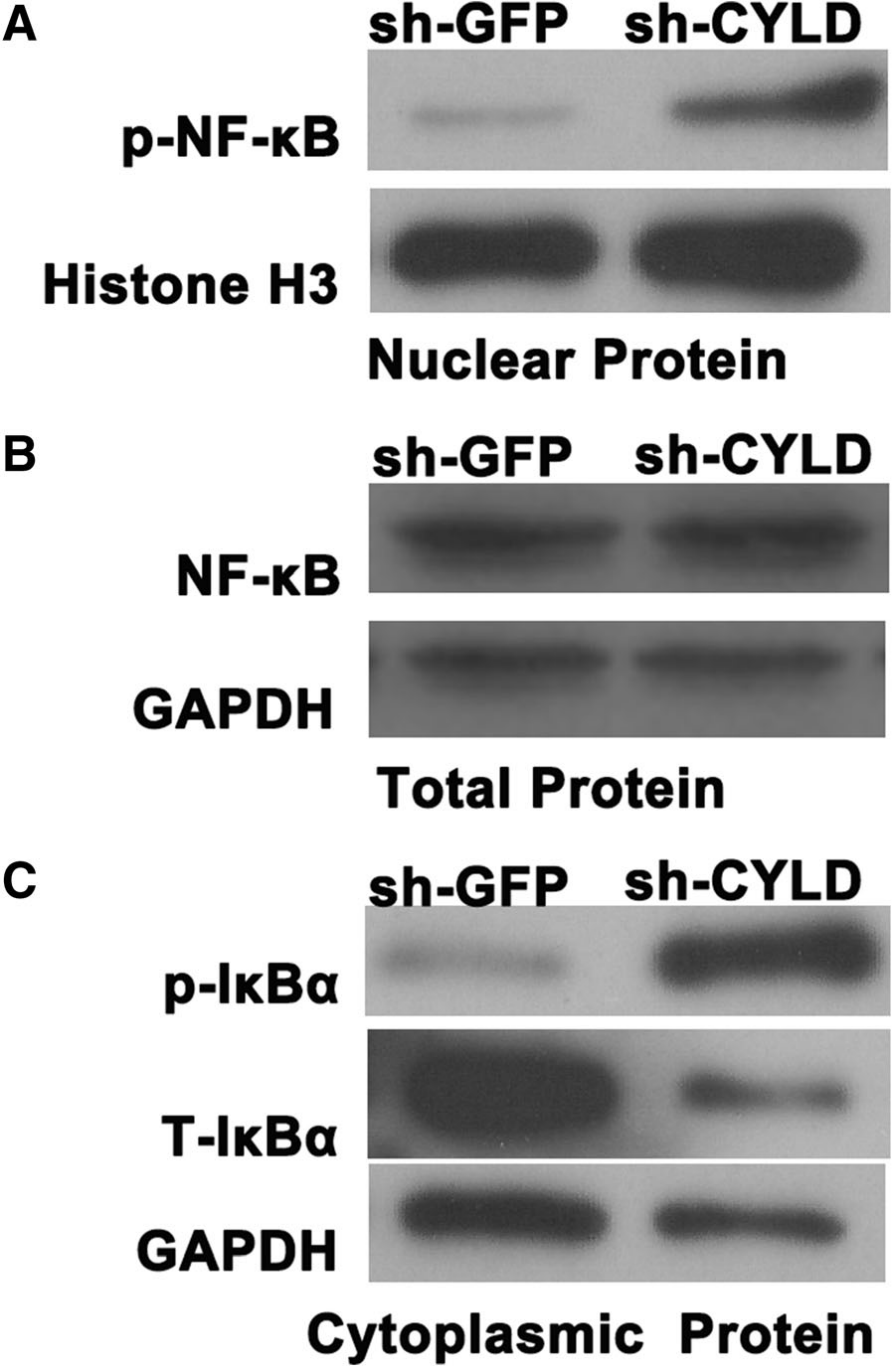
Effects of CYLD on NF-κB and IκBα activation in RA-FLSs. **a** After CYLD knockdown, we detected the protein level of NF-κB in RA-FLSs nuclei by Western blot. **b** After CYLD knockdown, we detected the protein level of NF-κB in RA-FLSs by Western blot. **c** After CYLD knockdown, we examined the protein level of p-IκBα and T-IκBα in RA-FLSs cytoplasm by Western blot

To explore the mechanism of CYLD on induction of pro-inflammatory cytokines, production of MMPs and RANKL, increased RA-FLS proliferation, decreased apoptosis, and cell cycle, we measured upstream NF-κB signaling by RT-PCR and Western blot. We showed that CYLD knockdown visibly aggravated NF-ΚB activity in RA-FLSs nuclei and IκBα in RA-FLSs cytoplasm and obviously decreased the level of total IκBα in RA-FLS cytoplasm (Fig. 6a), but not the total of NF-κB in RA-FLSs (Figs. 4a and 6b).

## Discussion

CYLD was initially reported as a tumor suppressor with deubiquitinating enzyme activity that is mutated in familial cylindromatosis. It suppresses the activation of NF-κB, which plays central roles in inflammation, immune responses, carcinogenesis, protection against apoptosis, and promotion of cell proliferation [21]. Our current study showed that CYLD expression in synovia from RA patients was significantly downregulated compared with that in synovia from OA patients. Conversely, NF-κB expression was distinctly upregulated in the RA synovium compared with the OA synovium. This result is consistent with that of a past study which found that NF-κB activation is higher in RA than in OA [36]. In addition, we found a significant inverse correlation between CYLD and NF-κB in the RA synovium, suggesting that NF-κB activation is suppressed when CYLD function is normal, but that it tends to be enhanced in the absence of functional CYLD. Moreover, synovial CYLD expression correlates significantly with ACPA, CRP, and RF. Above all, our results support the possibility that NF-κB activation accompanied by loss of CYLD may be a crucial step in the development and progression of RA. Thus, we postulated that functional relevant loss of CYLD expression may contribute to RA development and progression and may provide a new target for therapeutic strategies.

RA is a chronic inflammatory disease characterized by persistent inflammation of the diarthrodial joints with synovial hyperplasia and progressive joint destruction. In the terminal layer of the hyperplastic rheumatoid synovium, FLSs are the dominant cell types. Similar to tumor cells, they can be expanded in cell culture over several passages and escape contact inhibition to invade and degrade the adjacent cartilage and bone [37]. Activated RA-FLSs can be detected early after RA onset by visible erosions at cartilage–bone junctions [7].There, RA-FLSs can be activated by (among other things) inflammatory factors, TLR activation, matrix degradation products, and epigenetic modifications [8]. Recent studies have revealed that at an initial stage of synovial activation, TLRs are the key recognition structures of innate immune stimuli, such as microbial components, endogenous ligands, liberated cellular RNA, and DNA fragments from necrotic cells within the synovial fluid [38, 39]. TLRs are stimulated by their respective ligands; they dimerize and recruit downstream adaptor molecules, which propagate signals that converge at NF-κB through the TRAF6, TRAF3, and IL-1- and IL-1R-associated kinase 1/4 (IRAK1/4) complex and induce the production cytokines such as TNF-α, IL-1β, and IL-6 [40]. In addition, RA-FLS activation is not only the response to active inflammation within the synovium and the presence of inflammatory cells but constitutes an intrinsic feature of RA-FLSs. This inflammation-independent activation has been elegantly confirmed by studies in which human RA-FLSs implanted into a severe combined immunodeficient (SCID) mouse model of RA attached to and invaded co-implanted healthy human cartilage without cells of the immune system [41]. In addition, Murphy Roths Large mice with or without lymphoproliferation (MRL − lpr/lpr mice) can spontaneously develop RA-like arthritis, indicating that synovial cell proliferation and invasion into joint structures occur even before inflammatory cells migrate into the synovium [42]. Hence, RA-FLSs potentially contribute to the initiation and early progression of RA and are thus key factors in the pathogenesis of RA. Therefore, targeting the aggressive, joint-destructive, and pro-inflammatory properties of RA-FLSs is a recent approach to RA treatment.

Activated NF-κB plays a central role in regulating the expression of numerous genes during the inflammatory response, which is the fundamental process of RA. Generally, NF-κB is present in the cytoplasm as a heterodimeric complex composed of the p50 and p65 subunits, which are associated with inhibitory κB proteins (IκBs) [43]. NF-κB activation typically occurs following specific serine/threonine kinase IκB kinase (IKK) signalosome activation, leading to the phosphorylation and subsequent dispatch of IκBs to the proteasome of protein degradation [44]. IκB degradation enables NF-κB to translocate to the nucleus, stimulating the transcription of specific genes. The IKK complex consists of at least three subunits, including the kinases IKK-α and IKK-β and the associated regulatory subunit IKK-γ [44]. It has been demonstrated that NF-κB and IKK are abundant in RA-FLSs [26, 36]. NF-κB activation can robustly induce pro-inflammatory cytokines, MMPs, and RANKL production in RA-FLSs, which enhances the inflammatory cycle and contributes to joint erosion in RA [45, 46]. NF-κB has been shown to mediate fibronectin fragment-induced chondrocyte activation and to increase pro-inflammatory cytokines, chemokines, MMPs such as IL-6 and IL-8, and methyl-accepting chemotaxis protein-1 (MCP-1), growth-related oncogenes a, b, and g (*GRO-α*, *GRO-b*, and *GRO-g*, respectively), and MMP-13 production in human articular chondrocytes [47, 48]. TNF-α and IL-1β can strongly promote NF-κB activation and enhance the production of IL-6, IL-8, intercellular adhesion molecule 1 (ICAM-1), and collagenase in RA-FLSs [49]. TNF-α, IL-1β, and IL-6 are prominent pro-inflammatory cytokines in the inflammatory cascade of RA and have been widely studied as therapeutic interventions [5, 50].

Numerous studies in vitro and in vivo have validated that CYLD mediates NF-κB activation by deubiquitinating TRAF2, TRAF6, and NEMO, making it an important regulator in the adaptive immune response. However, the expression and exact roles of CYLD in the pro-inflammatory effects of RA-FLSs are unknown. We observed CYLD expression in the lining and sublining layers of RA synovium, with intense staining found in the endochylema as well as the nuclei of lining cells (both macrophage-like synoviocytes and FLSs) and sublining of inflammatory cells, implying that CYLD was expressed in RA-FLSs. Its expression in primarily cultured RA-FLSs was significantly lower than that in OA-FLSs. Our experiments also further demonstrated that suppression of CYLD by sh-CYLD enhanced mRNA expression and the secretion of pro-inflammatory cytokines in RA-FLSs, including TNF-α, IL-1β, IL-6, and IL-8; aggravated the activity of NF-κB in RA-FLS nuclei and IκBα in RA-FLS cytoplasm; and decreased the level of total IκBα in RA-FLS cytoplasm. This suggests that downregulated CYLD plays a conspicuous role in the pro-inflammatory effects of RA-FLSs via NF-κB signaling.

In joints with early-stage RA, activated RA-FLSs attach to and overgrow the articular cartilage surface, then invade and destroy cartilage and induce bone resorption via secretion of MMPs, cathepsins, and inflammatory cytokines and regulation of monocyte-to-osteoclast differentiation [51, 52]. MMPs include collagenases (MMP-1, MMP-8, and MMP-13), gelatinases (MMP-2, MMP-9), stromelysins (MMP-3, MMP-7, MMP-10, MMP-11, and MMP-12), and membrane-type (MT) MMPs (MT1-MMP, MT2-MMP, MT3-MMP, and MT4-MMP). In situ hybridization studies have validated that lining cells are the major source of MMP-1 and MMP-3 in the synovium, especially RA-FLSs [53, 54]. MMP-1 cleaves collagens I, II, VII, and X, while inhibition of MMP-1 synthesis significantly reduces the cartilage invasiveness of FLSs in the SCID mouse model for RA [55]. MMP-3 can degrade cartilage proteoglycans and type IX collagen and activate other pro-MMPs, including pro-MMP-1 and pro-MMP-9 [56]. In addition, osteoclasts are the major course of local and systemic abnormalities of bone remodeling, including bone erosion and both focal and systemic osteoporosis [57]. RANKL is one of the key molecules for osteoclastic differentiation and activity that have been found to be strongly expressed at sites of bone erosion in RA synovium, especially in RA-FLSs [58]. Studies performed in human RA-FLSs have indicated that IL-1β mediates the production of MMP-1 and MMP-3 by regulating NF-κB activation [59]. Inhibition of NF-κB and Jun N-terminal kinase (JNK) activation significantly decreases production of pro-inflammatory cytokines (TNF-α, IL-1β, IL-6, and IL-8) and MMPs (MMP-1 and MMP-3) in RA-FLSs [60]. It has also been reported that NF-κB suppression obviously reduces RANKL expression in human RA-FLSs and in FLSs from mice with adjuvant-induced arthritis (AA) [61, 62]. As CYLD plays a pivotal role in regulating NF-κB activation, it is not clear whether it is involved in the synthesis of MMP-1, MMP-3, and RANKL in RA-FLSs. We showed that CYLD inhibition in RA-FLSs apparently enhanced MMP-1, MMP-3, and RANKL production and NF-κB activity, implying that CYLD participated in mediating RA-FLS-induced cartilage and bone destruction via NF-κB signaling.

In the pathology of RA, it is widely acknowledged that the progressive destruction of articular cartilage relies on the evolution of hyperplastic synovial tissue, while hyperplasia in FLSs depends on dysregulated proliferation and apoptosis [63]. Thus, promoting RA-FLS apoptosis and reducing RA-FLS proliferation are new treatment strategies for RA. It has been reported that CYLD attenuates NF-κB signaling by removing lysine63 (Lys63)-linked polyubiquitin chains from TRAF2 or TRAF6, leading to programmed cell death [64]. Previous studies have also revealed that CYLD suppression leads to decreased apoptosis and increased proliferation by activating NF-κB [23, 65]. In the present study, we found that CYLD inhibition distinctly enhanced proliferation, reduced apoptosis, and increased the cell division of RA-FLSs and aggravated the activity of NF-κB in these cells, suggesting that CYLD is involved in RA-FLS hyperproliferation in RA. However, a recent report indicated that the loss of CYLD in mouse keratinocytes enhances cell proliferation by increasing the nuclear activity of Bcl-30-associated NF-κB p50 and p52 subunits, rather than by increasing classical p65/p50 NF-κB action. This implies that the mechanism of CYLD-mediated NF-κB inhibition may vary by cell type [65].

## Conclusions

In summary, downregulated CYLD may be involved in the pathogenesis of inflammation in the RA synovium by regulating classical NF-κB activation, as well as in the pro-inflammatory effects and hyperproliferation of RA-FLSs. Thus, CYLD may provide a potential target for the treatment of RA. However, the precise mechanism of interaction between CYLD and NF-κB in RA-FLSs remains unclear and requires further exploration. In addition, our study showed that CYLD is expressed not only in RA-FLSs but also in inflammatory cells, such as lymphocytes, and in plasma cells. Further studies are needed to investigate the expression and role of CYLD in regard to the pro-inflammatory effects, proliferation, apoptosis, and cell cycles of these cells.

## Abbreviations

28SJC: Swollen joint count of 28 joints
28TJC: Tender joint count of 28 joints
7-AAD: 7-aminoactinomycin D
AA: Adjuvant-induced arthritis
ACPA: Anti-cyclic citrullinated peptide antibody
ACR: American College of Rheumatology
ANOVA: Aanalysis of variance
BCA: Bicinchoninic acid
Bcl-2: B-cell lymphoma 2
BSA: Bovine serum albumin
CCK-8: Cell Counting Kit-8
CDAI: Clinical Disease Activity Index
cDNA: Complementary deoxyribonucleic acid
CRP: C-reactive protein
CYLD: Cylindromatosis
DAB: 3,3′-Diaminobenzidine
DAPI: 4,′6-diamindino-2-phenylindole
DAS28 [4]-CRP: DAS28 with 4 variables, including CRP
DAS28: Disease Activity Score 28-joint assessment
DMARDs: Disease-modifying anti-rheumatic drugs
DMEM/F12: Dulbecco’s modified Eagle’s medium–Ham’s F-12
ECL: Enhanced chemiluminescence
ECM: Extracellular matrix
ELISA: Enzyme-linked immunosorbent assay
ESR: Erythrocyte sedimentation rate
EULAR: European League Against Rheumatism
FACS: Fluorescence-activated cell sorting
FBS: Fetal bovine serum
FCM: Flow cytometry
FLIP: Fas-associated death domain-like interleukin-1-converting enzyme inhibitory protein
FLS: Fibroblast-like synoviocyte
GAPDH: Glyceraldehyde 3-phosphate dehydrogenase
*GRO-α*: Growth-related oncogene-alpha
H&E: Hematoxylin and eosin
HAQ: Chinese-language version of Stanford Health Assessment Questionnaire
HRP: Horseradish peroxidase
ICAM-1: Intercellular adhesion molecule 1
IgG: Immunoglobulin G
IKK: IκB kinase
IL-1β: Interleukin-1 beta
IL-6: Interleukin-6
IL-8: Interleukin-8
IRAK1/4: IL-1- and IL-1R-associated kinase 1/4
IκB: Inhibitory κB protein
IκBα: Nuclear factor of κ-light polypeptide gene enhancer in B-cells inhibitor-α
LR: Early apoptosis
Lys63: Lysine63
MCP-1: Methyl-accepting chemotaxis protein
MMP: Matrix metalloproteinase
MRL − lpr/lpr mice: Murphy Roths Large mice with or without lymphoproliferation
mRNA: Messenger ribonucleic acid
MT: Membrane type
*n*: Number of patients
NEMO: NF-κB essential modulator
NF-κB: Nuclear factor κ-light-chain-enhancer of activated B cells
OA: Osteoarthritis
OD: Optical density
Pain VAS: Pain visual analog scale
PBS: Phosphate-buffered saline
PI: Propidium iodide
PrGA: Provider global assessment of disease activity
PtGA: Patient global assessment of disease activity
RA: Rheumatoid arthritis
RANKL: receptor activator of nuclear factor kappa-B ligand
RF: Rheumatoid factor
RNase: Ribonuclease
RT-PCR: Reverse transcription polymerase chain reaction
RT-qPCR: Quantitative reverse transcription polymerase chain reaction
SCID: Severe combined immunodeficiency
SD: Standard deviation
SDAI: Simple Disease Activity Index
SDS-PAGE: Sodium dodecyl sulfate-polyacrylamide gel electrophoresis
sh-CYLD: Lentiviral CYLD shRNA
sh-GFP: Lentiviral vector
shRNA: Short hairpin ribonucleic acid
SNP: Sodium nitroprusside
SUMO-1: Sentrin-1
Tak1: TGF-β-activated kinase 1
TBST: Tris-buffered saline-Tween
TLR: Toll-like receptor
TNF-α: Tumor necrosis factor alpha
TR: Total apoptosis
TRAF2: TNF receptor-associated factor 2
TRAF6: TNF receptor-associated factor 6
UR: Late apoptosis
